# Representing Dutch Older Adults During COVID-19: What Can We Learn?

**DOI:** 10.1093/geroni/igab046.363

**Published:** 2021-12-17

**Authors:** Jolanda Lindenberg, Miriam Verhage

**Affiliations:** 1 Leyden Academy on Vitality and Ageing, Leiden, Zuid-Holland, Netherlands; 2 Leyden Academy on Vitality and Ageing, Leyden Academy on Vitality and Ageing, Zuid-Holland, Netherlands

## Abstract

From the very beginning of the COVID-19 pandemic older adults have been at the heart of public debate. Early articles argued that public representation of older persons displayed a a resurgence of ageist stereotypes and beliefs in (inter)national media (e.g. Ayalon et al. 2020, Fraser et al 2020, Lichtenstein 2020, Sotomayer et al. 2020). Yet studies confirming this are absent up to now. In this paper, we present findings on the representation of Dutch older adults during the first six months of the COVID-19 crisis in The Netherlands. We analysed 1141 articles about older adults of the five largest newspapers using quantitative content analyses and discourse analysis to systematically explore patterns, sentiments and meaning in the articles. We show that the majority of these articles were published in general news and that older adults were rarely (2%) cited in these articles. Most prominent adjectives were vulnerable and weak. Most prominent substantives were attention, long-term care facility and loneliness. The sentiment was largely negative. Additionally, we find three discursive frames predominate: ‘an older people’s disease’, ‘vulnerability’ and ‘solidarity’. This evidences that the Dutch reporting on older adults during COVID-19 reproduced a discourse of dependency highlighting and further emphasizing the sociopolitical context before COVID-19 while drawing out earlier ageist tendencies. On the basis of our findings and drawing on advisory experiences, we discuss implications for policy, education and practice and how we can reframe and differently address older adults specifically in terms of language and their more (un)conscious positioning in (public) debate.

